# Protective effects of *Scoparia dulcis* L. extract on high glucose-induced injury in human retinal pigment epithelial cells

**DOI:** 10.3389/fnut.2023.1085248

**Published:** 2023-03-30

**Authors:** Heng-Dao Lin, Yuan-Chieh Lee, Chien-Yi Chiang, Yu-Jung Lin, Cheng Yen Shih, Rong-Kung Tsai, Pi-Yu Lin, Shinn-Zong Lin, Tsung-Jung Ho, Chih-Yang Huang

**Affiliations:** ^1^Cardiovascular and Mitochondrial Related Disease Research Center, Hualien Tzu Chi Hospital, Buddhist Tzu Chi Medical Foundation, Hualien, Taiwan; ^2^Department of Ophthalmology, Buddhist Tzu Chi General Hospital, Hualien, Taiwan; ^3^Department of Ophthalmology and Visual Science, Tzu Chi University, Hualien, Taiwan; ^4^Institute of Medical Science, Tzu Chi University, Hualien, Taiwan; ^5^Department of Ophthalmology, National Taiwan University Hospital, Taipei, Taiwan; ^6^Buddhist Compassion Relief Tzu Chi Foundation, Hualien, Taiwan; ^7^Institute of Eye Research, Hualien Tzu Chi Hospital, Buddhist Tzu Chi Medical Foundation, Hualien, Taiwan; ^8^Institute of Medical Sciences, Tzu Chi University, Hualien, Taiwan; ^9^Taiwan Buddhist Tzu-Chi Foundation, Hualien, Taiwan; ^10^Buddhist Tzu Chi Bioinnovation Center, Buddhist Tzu Chi Medical Foundation, Hualien, Taiwan; ^11^Department of Neurosurgery, Hualien Tzu Chi Hospital, Hualien, Taiwan; ^12^Integration Center of Traditional Chinese and Modern Medicine, Hualien Tzu Chi Hospital, Hualien, Taiwan; ^13^Department of Chinese Medicine, Hualien Tzu Chi Hospital, Hualien, Taiwan; ^14^School of Post-Baccalaureate Chinese Medicine, College of Medicine, Tzu Chi University, Hualien, Taiwan; ^15^Department of Medical Laboratory Science and Biotechnology, Asia University, Taichung, Taiwan; ^16^Graduate Institute of Biomedical Sciences, China Medical University, Taichung, Taiwan; ^17^Center of General Education, Buddhist Tzu Chi Medical Foundation, Tzu Chi University of Science and Technology, Hualien, Taiwan; ^18^Department of Medical Research, China Medical University Hospital, China Medical University, Taichung, Taiwan

**Keywords:** retinal pigment epithelium, diabetic retinopathy, high glucose, traditional Chinese medicine, ROS, inflammation

## Abstract

Diabetic retinopathy (DR) is a major cause of vision loss in diabetic patients. Hyperglycemia-induced oxidative stress and the accumulation of inflammatory factors result in blood-retinal barrier dysfunction and the pathogenesis of DR. *Scoparia dulcis* L. extract (SDE), a traditional Chinese medicine, has been recently recognized for its various pharmacological effects, including anti-diabetic, anti-hyperlipidemia, anti-inflammatory, and anti-oxidative activities. However, there is no relevant research on the protective effect of SDE in DR. In this study, we treated high glucose (50 mM) in human retinal epithelial cells (ARPE-19) with different concentrations of SDE and analyzed cell viability, apoptosis, and ROS production. Moreover, we analyzed the expression of Akt, Nrf2, catalase, and HO-1, which showed that SDE dose-dependently reduced ROS production and attenuated ARPE-19 cell apoptosis in a high-glucose environment. Briefly, we demonstrated that SDE exhibited an anti-oxidative and anti-inflammatory ability in protecting retinal cells from high-glucose (HG) treatment. Moreover, we also investigated the involvement of the Akt/Nrf2/HO-1 pathway in SDE-mediated protective effects. The results suggest SDE as a nutritional supplement that could benefit patients with DR.

## Introduction

Diabetic retinopathy (DR) is the most common and serious complication of diabetes mellitus (DM), which can lead to vision loss in diabetes patients and seriously degrade their quality of life (1). It has been reported that nearly 35.4% of diabetes patients demonstrate different forms of diabetic retinopathy (DR) (2). The leading contributor to the development of DR is hyperglycemia (3). In addition, excessive blood sugar gives rise to the deposition of atherosclerotic plates, which in turn provokes inflammation of the retinal vessels (4). The currently dominant treatment approach available for DR is the control of microvascular complications, including intravitreal drug therapy, laser photocoagulation, vitreous surgery, and anti-vascular endothelial growth factor (VEGF) molecules (5, 6). However, these treatment approaches are only effective for advanced DR. As the prevalence of diabetes increases, new approaches are needed for the treatment or prevention of DR in earlier stages. To avoid making DR one of the major burdens on public health, it is necessary to establish novel and effective strategies to prevent and treat it in the early stage.

In recent years, human retinal epithelial cells (ARPE-19) have been widely used as *in vitro* models for DR research (7, 8). The retinal pigment epithelium (RPE) is a single layer of epithelial cells located between the vascular choroids and the neurosensory retina. The RPE performs different functions, including turnover of photoreceptor outer segments and oxidative stress response, and forms the protected outer blood-retinal barrier, which maintains the normal structure and function of the retina (9). It has been reported that dysfunction of RPE is one of the early events that occur before vision loss or DR (10, 11). Many studies have reported that oxidative stress and inflammation triggered by high glucose are crucial in the pathogenesis of retinopathy (12, 13). Furthermore, chronic hyperglycemia-induced ROS production leads to RPE dysfunction and eventually destroys the barrier function, resulting in angiogenesis (14). The damage to cells causing the accumulation of reactive oxygen species (ROS) further enhances lipid peroxidation such as 4-HNE and 4-HDDE (15). Hence, we can reasonably consider that hyperglycemia-induced oxidative stress in RPE plays a critical role in DR.

Recently, traditional Chinese medicine (TCM) has been a hot topic in clinical applications and experimental studies for DR treatment, due to its low price and fewer side effects (16). In recent decades, *Scoparia dulcis* L., a member of the plantain family that is employed in traditional Chinese medicine, has been widely used for clinical purposes in China, Korea, Japan, and other Asian countries to contribute to stomachic, diuretic, anti-tussive, heat-clearing, and toxin-absorbing effects (17). Many studies have indicated that *Scoparia dulcis* L. provides various pharmacological effects, for example, the treatment of metabolic syndromes, including anti-diabetic, anti-hyperlipidemia, anti-inflammatory, anti-atherosclerotic, anti-arthritic, hepatoprotective, anti-oxidative, and anti-urolithiasis activities (18). *Scoparia dulcis* L. contains numerous constituents, such as flavonoids and polysaccharides, and each specific component has been reported to possess different degrees of anti-oxidant activity (19). In addition, some animal studies have also demonstrated that *Scoparia dulcis* L. exhibits anti-diabetic and anti-oxidant effects in mice or rats (20, 21). Since SDE exhibits significant anti-diabetic effects such as hypoglycemic activity, insulin mimetic activity, and anti-oxidant effects, we reasonably hypothesized that SDE could have potential effects on complications of diabetes. However, there is no study on the protection of SDE in glycemia-related retinopathy. In the present study, we used ARPE-19 as an *in vitro* model to determine the effects of SDE on HG conditions and elucidate its possible protective mechanisms. Our results demonstrate that SDE protects ARPE-19 cells from HG-induced oxidative stress, inflammation, and cell apoptosis, which may provide new insights into the treatment of DR.

## Materials and methods

### Cell cultures and treatment

The human retinal pigmented epithelium cell line ARPE-19 (BCRC number 60383) was obtained from the Food Industry Research and Development Institute (FIRDI, Hsinchu, Taiwan). These cells were maintained in a 1:1 mixture of DMEM/F-12, supplemented with 10% fetal bovine serum (FBS, GIBCO) and 1% penicillin/streptomycin (PS, Gibco) in a humidified 5% CO_2_ incubator at 37°C. To differentiate ARPE-19 cells to a more native and physiologically relevant state, once confluent, the media was switched to a specialized DMEM media that contained high glucose (4.5 g/l) supplemented with 1% heat-inactivated FBS, 1 mM sodium pyruvate (Hyclone),2 mM L-glutamine (Hyclone), and 10 mM nicotinamide (Sigma) for 2 months. In all cases, media exchange was performed three times a week. Before treatment, ARPE-19 cells were seeded into 96-well plates or 6 cm Petri dishes at a density of 5 × 10^4^ cells/mL and incubated overnight. After overnight culture, the culture medium was replaced with a serum-free medium and treated with different concentrations (25, 50, 100, and 200 μg/ml) of *Scoparia dulcis* L. extract (SDE) for 24 h. Following this, the cells were treated with 50 mM D-glucose (Sigma) for an additional 48 h. β-cyclocitral (Sigma), 1-methyl-2-pyrrolidinone (Sigma), procaine (Sigma), cyclohexylamine (Sigma), and N1- acetylspermine (Sigma) are used as the standard for HPLC–MS analysis.

### Preparation of *Scoparia dulcis* L. extract

Dry *Scoparia dulcis* L. (60 g) was boiled with 600 ml RO water and concentrated to 60 ml. Then, supernatant (5 ml) was collected and dried by a freeze dryer and stored at −20°C. The stock solution of the Scoparia dulcis L. extract was prepared with dd H_2_O to a concentration of 0.05 g/ml. The stock solution was filtered and sterilized by a 0.22 μM microporous membrane before cell treatment.

### LC–MS/MS analysis

*Scoparia dulcis* L. extract was filtrated with a 0.45 μm microporous membrane to obtain the test solution and 1 ml. Three baths of SDE samples were sent to a biotechnology company (PRO TECH) for HPLC and MS analysis. A total of 100 μl SDE and 400 μl acetonitrile were vortexed to mix, incubated at −20°C for 30 min, and centrifuged at 15000 g for 10 min. All analyses were performed on the Agilent 1,260 HPLC system with Phenomenex Luna HILIC-200 A column (50 mm × 2.0 mm i.d., 3.0 um) and AB Sciex Instruments QTRAP 5500.

### Cell viability assay

CCK-8 assay was used to determine the cell viability of ARPE-19 cells, which were treated with various concentrations of SDE in the absence or presence of 50 mM D-glucose for 24 h. Thereafter, for the culture termination, 10 μl of CCK-8 was added to each well and then incubated for 2 h at 37°C in a 5% CO_2_ incubator. Finally, the absorbance of each well was recorded at 450 nm using a microplate reader (Thermo).

### Apoptosis assay

Cell apoptosis was determined by the Terminal Deoxynucleotidyl Transferase dUTP Nick End Labeling (TUNEL) assay. TUNEL was performed using a commercial kit (*In Situ* Cell Death Detection Kit, Roche) according to the manufacturer’s instructions, as in our previous study (22). The 8-well chamber slides were fixed with 4% paraformaldehyde for 15 min at room temperature and permeabilized with 0.5% Triton X-100 for 10 min. After three rinses with PBS, 100 μl of the TUNEL reaction mixture was added, and the slides were incubated in a humidified atmosphere for 1 h at 37°C in the dark. The slides were analyzed under a fluorescence microscope (Olympus) with an excitation wavelength of 488 nm.

### *In situ* senescence-associated acid beta-galactosidase assay

β-Galactosidase activity is used as a biomarker for senescent and aging cells. Here, we used the SPiDER-ßgal kit (Dojindo Molecular Technologies), which was reconstituted in 20 μl dimethyl sulfoxide to detect cell β-Galactosidase activity. Before the senescence assay, SPiDER-ßgal was reconstituted in 20 μl dimethyl sulfoxide (DMSO, Sigma). We washed the cells three times with PBS and then incubated them with 1:500 dilution SPiDER-βGal for 30 min. After that, the cells were fixed with 4% paraformaldehyde for 15 min at room temperature. The slides were analyzed under a fluorescence microscope (Olympus).

### Measurement of intracellular ROS level and mitochondrial superoxide

Intracellular reactive oxygen species (ROS) generation was detected in living cells by a DCF-DA kit, and mitochondrial superoxide was detected by fluorogenic dye MitoSOX (Invitrogen). We seeded cells in an 8-well chamber slide at a density of 5 × 10^4^ cells/mL and incubated them for 24 h at 37°C. We washed them three times with PBS and added 10 μM of chloromethyl 2′,7′-dichlorodihydrofluorescein diacetate (CM-H_2_DCFDA) for 20 min or 5 μM mitoSOX for 10 min to the cells in the dark. After incubation, we washed the cells gently three times with PBS and added 1 μg/ml Hoechst staining solution (Thermofisher) to them. These cells were observed by use of fluorescence microscopy. The acquired images were converted into binary images for the quantification of the average fluorescence intensity using ImageJ software. We set the related fluorescence unit of the control group as 1. We repeated each experiment three times.

### Immunofluorescence staining

The ARPE-19 cells were seeded into an 8-well chamber slide (SPL) and incubated overnight. They were treated with various concentrations of SDE (25, 50, and 100 μg/ml) for 24 h and then treated with D-Glucose (50 mM) for 48 h. After treatment, the cells were fixed with 4% paraformaldehyde for 15 min and then permeabilized with 0.1% Triton X-100 for 10 min. The cells were washed with PBS and blocked with 2% bovine serum albumin (BSA) in PBS for 1 h. Appropriate primary anti-bodies were incubated with 2% BSA overnight (Supplementary Table S1) and then with a fluorescein (FITC) (488 nm or 594 nm)-conjugated secondary anti-body for 45 min in 2% BSA. The cells were later stained with 1 μg/ml of DAPI for 5 min, washed with PBS three times, and then subjected to image acquisition by fluorescence microscopy (Olympus). The relative density of immunostaining was analyzed by ImageJ, comparing ARPE-19 cells treated with different SDE concentrations in the high-glucose treatment group with those with a low-glucose condition (as a normal reference).

### Western blot

We conducted a Western blot analysis following the procedure, as described in our previous study (23, 24). Briefly, we separated protein samples (30–40 μg) using 9–12% SDS-PAGE (Bio-Rad), which we then transferred to polyvinylidene fluoride (PVDF) membranes. The membranes were blocked with Tris-buffered saline (TBS) containing 2% BSA and 0.05% Tween 20 for 1 h at room temperature. After three washes in TBS containing 0.05% Tween 20 (TBST), we incubated the membranes with appropriate primary anti-bodies at 4°C overnight (Supplementary Table S1). After three washes in TBST, we incubated the membranes with relevant secondary anti-bodies (anti-mouse or anti-rabbit HRP-linked, Cell Signaling) at room temperature for 45 min. After three times washes in TBST, we added the membranes to chemiluminescent HRP (Millipore). We used ImageJ to measure the band density and used GAPDH as an internal reference protein in each group.

### Catalase activity assay

The ARPE-19 cells were pretreated with or without various SDE concentrations (25, 50, 100, and 200 μg/ml) in a 6 cm Petri dish for 24 h. Then, D-glucose (50 mM) was added, and the cells were cultured for 48 h. A catalase activity ELISA kit (Catalase assay kit, Cayman, United Kingdom, 707002) was used to analyze the catalase change. Briefly, the treatment cells were harvested, and the homogenies were followed using the manufacturer’s instructions. The OD at 540 nm was determined using a microplate reader (Thermo).

### IL-6, IL-8 MCP-1 ELISA assay

The ARPE-19 cells were pretreated with or without various SDE concentrations (25, 50, 100, and 200 μg/ml) in 96-well plates for 24 h. Then, D-glucose (50 mM) was added, and the cells were cultured for 48 h. Specific ELISA kits (EZIL6-98 K, EZHIL8-100 K, EZMCP1-99KRM; Merck Millipore) were used to measure the levels of interleukin-6 (IL-6), interleukin-8 (IL-8), and monocyte chemoattractant protein-1 (MCP-1) in the supernatants, following the manufacturers’ instructions. The OD at 450 nm was determined using a microplate reader (Thermo).

### Statistical analysis

We presented the data as the mean ± standard deviation for at least three different experiments. All the statistical analyses were performed by GraphPad Prism 6 software, and one-way ANOVA was used for analyzing the data. A statistically significant difference was considered as *p* < 0.05 and is shown as follows: *: 0.01 < *p* ≤ 0.05, **: *p* ≤ 0.01.

## Results

### Identification of the components of *Scoparia dulcis* L. extract

To standardize the chemical composition of the SDE, we performed HPLC and MS analysis. According to Wankhar et al. we selected β-cyclocitral, 1-methyl-2-pyrrolidinone, procaine, cyclohexylamine, and N1-acetylspermine as the standards to analyze the constituents of SDE (19). The chromatogram of the compounds obtained is shown in Supplementary Figure S1 and Supplementary Table S2.

### *Scoparia dulcis* L. extract prevents high-glucose-mediated cellular death and senescence

**Figure 1:**
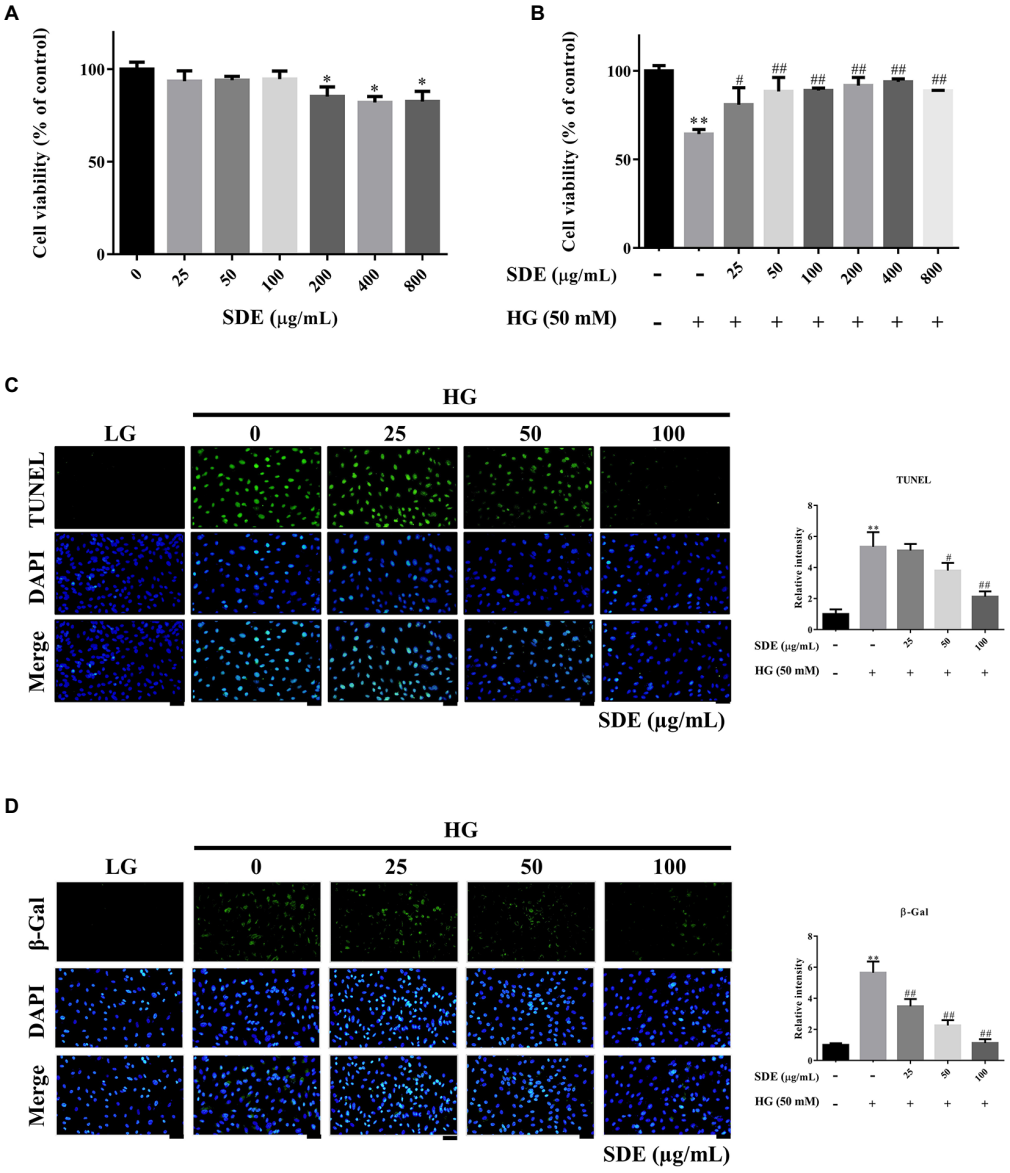
Effect of *Scoparia dulcis* L. extract (SDE) and glucose on the viability and apoptosis of ARPE-19 cells. **(A)** The ARPE-19 cells were treated with various concentrations (25 ~ 800 μg/ml) of SDE for 24 h, and the cell viability was analyzed by CCK-8 assay. **(B)** The effect of SDE (25 ~ 800 μg/ml) on the viability of ARPE-19 cells treated with high-glucose (HG-50 mM). **(C)** Cell apoptosis was determined by TUNEL assay. **(D)** SA-β-gal staining was used to detect the senescent cells. All results are representative of three independent experiments, and values are mean ± SD. Scale bars, 50 μm. (*p < 0.05, **p < 0.01 vs. control and ^#^*p* < 0.05, ^##^*p* < 0.01 for HG vs. HG plus SDE).

First, we evaluated the effect of SD extract on the ARPE-19 cells by incubating with a series of concentrations of SD extract (0, 25, 50, 100, 200, 400, and 800 μg/ml). The CCK-8 assay showed that administration of SDE had a significant effect on cell viability; however, the viability of the ARPE-19 cells was unaffected by SDE at concentrations of 25, 50, and 100 μg/ml (Figure 1A). Thus, 25–100 μg/ml of SDE was used in the following experiments. Then, we investigated the effect of SD extract on cell viability in HG-induced ARPE-19 cells. HG caused a significant decrease in the cell viability of the ARPE-19 cells, while ARPE-19 treatment reversed the inhibitory effect of HG stimulation on cell viability (Figure 1B). We further investigated whether SDE inhibited HG-mediated cellular apoptosis. As shown in Figure 1C, SDE dose-dependently decreased cell apoptosis compared with the HG group. Figure 1D also shows that SDE significantly inhibited HG-induced β-galactosidase expression, indicating that SDE can prevent cell senescence from HG treatment. Taken together, these results suggest that SDE protects ARPE-19 cells from HG-stimulated cell apoptosis and senescence.

### *Scoparia dulcis* L. extract inhibits oxidative stress and oxidative damage in ARPE-19 cells exposed to HG

**Figure 2:**
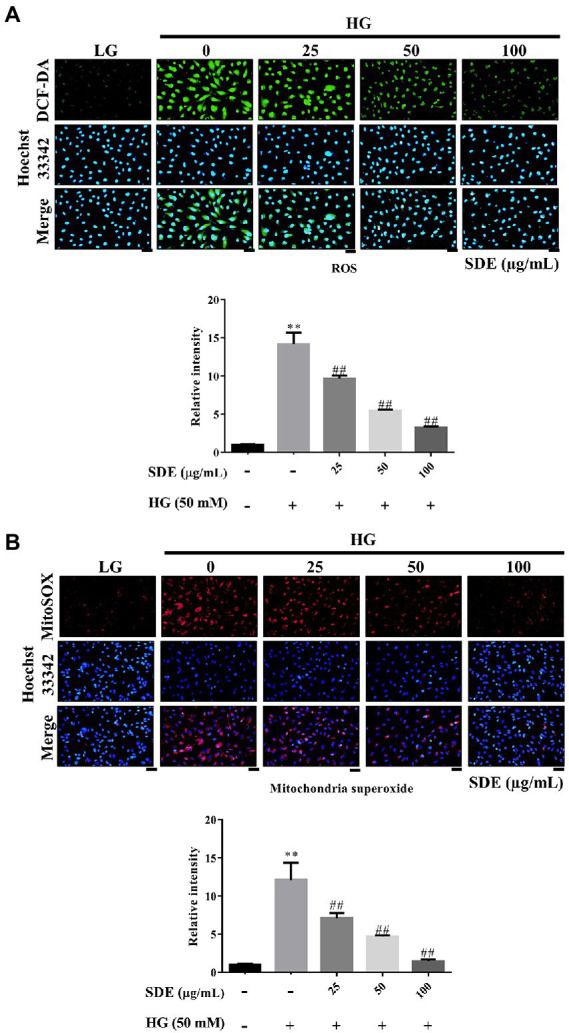
Effect of *Scoparia dulcis* L. extract (SDE) on anti-oxidative stress with glucose treatment on ARPE-19 cells. The ARPE-19 cells were treated with SD extract (25, 50, 100, or 200 μg/ml) for 24 h, and following this, they were treated with 50 mM glucose for 24 h. **(A)** The intracellular ROS levels were measured by DCF-DA staining. **(B)** The mitochondria superoxide was measured by MitoSOX red. Scale bars, 50 μm. LG, low glucose; HG, high glucose; SDE, *Scoparia dulcis* L. extract. All results are representative of three independent experiments, and values are mean ± SD. Scale bars, 50 μm. (**p < 0.01 vs. control and ^##^*p* < 0.01 for HG vs. HG plus SDE).

**Figure 3:**
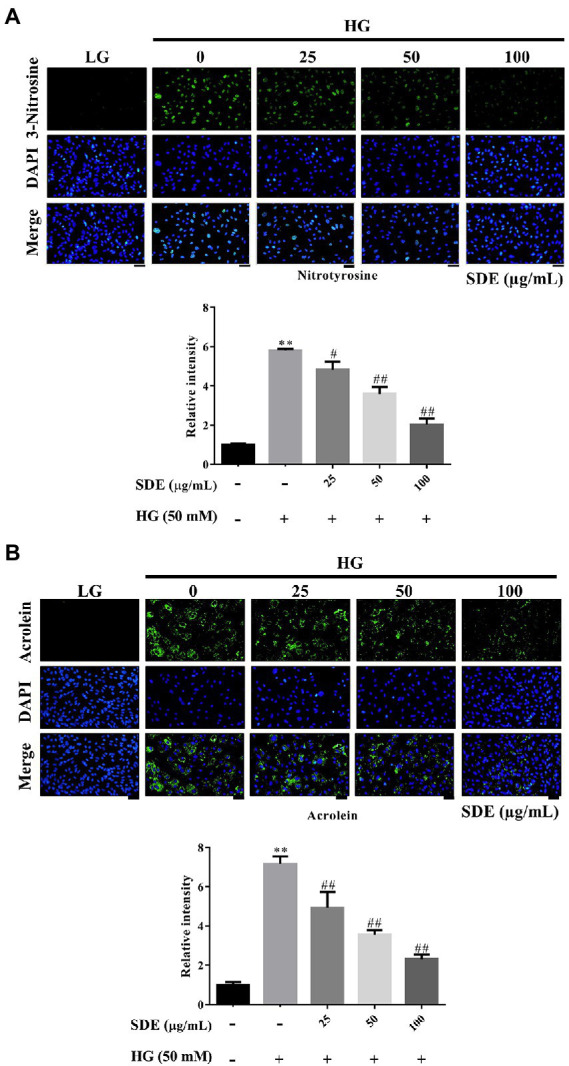
Effect of *Scoparia dulcis* L. extract (SDE) on anti-oxidative stress product with high-glucose treatment on ARPE-19 cells. The ARPE-19 cells were treated with SDE (25, 50, 100, or 200 μg/ml) for 24 h, and following this, they were treated with 50 mM glucose for 48 h. **(A)** Nitrotyrosine and **(B)** acrolein were detected using immunofluorescence (IF) imaging. The relative fluorescence signals of the IF image were quantified using ImageJ software. Scale bars, 50 μm. DAPI, 4′,6-diamidino-2- phenylindole; LG, low glucose; HG, high glucose; SDE, *Scoparia dulcis* L. extract. All results are representative of three independent experiments, and values are mean ± SD. Scale bars, 50 μm (**p < 0.01 vs. control and ^#^*p* < 0.05, ^##^*p* < 0.01 for HG vs. HG plus SDE).

To evaluate the effect of SDE on HG-stimulated oxidative stress and oxidative damage, we detected the changes in ROS production and ROS-related protein (3-Nitrosine) or lipid (Acrolein) damage. As shown in Figure 2A, after exposure to HG, ROS production was significantly increased in the ARPE-19 cells, and SDE treatment resulted in significant decreases in ROS production. In addition, we also found SDE can significantly decrease mitochondria superoxide with HG treatment (Figure 2B). As indicated in Figure 3, the HG significantly induced protein and lipid damage. Our results showed that SDE not only reduced oxidative protein product (Figure 3A) but also reduced oxidative lipid product (Figure 3B). These results indicated that SD extract can inhibit ROS production and alleviate oxidative damage with HG treatment.

### *Scoparia dulcis* L. extract reduces ROS through upregulation of anti-oxidative enzymes

**Figure 4:**
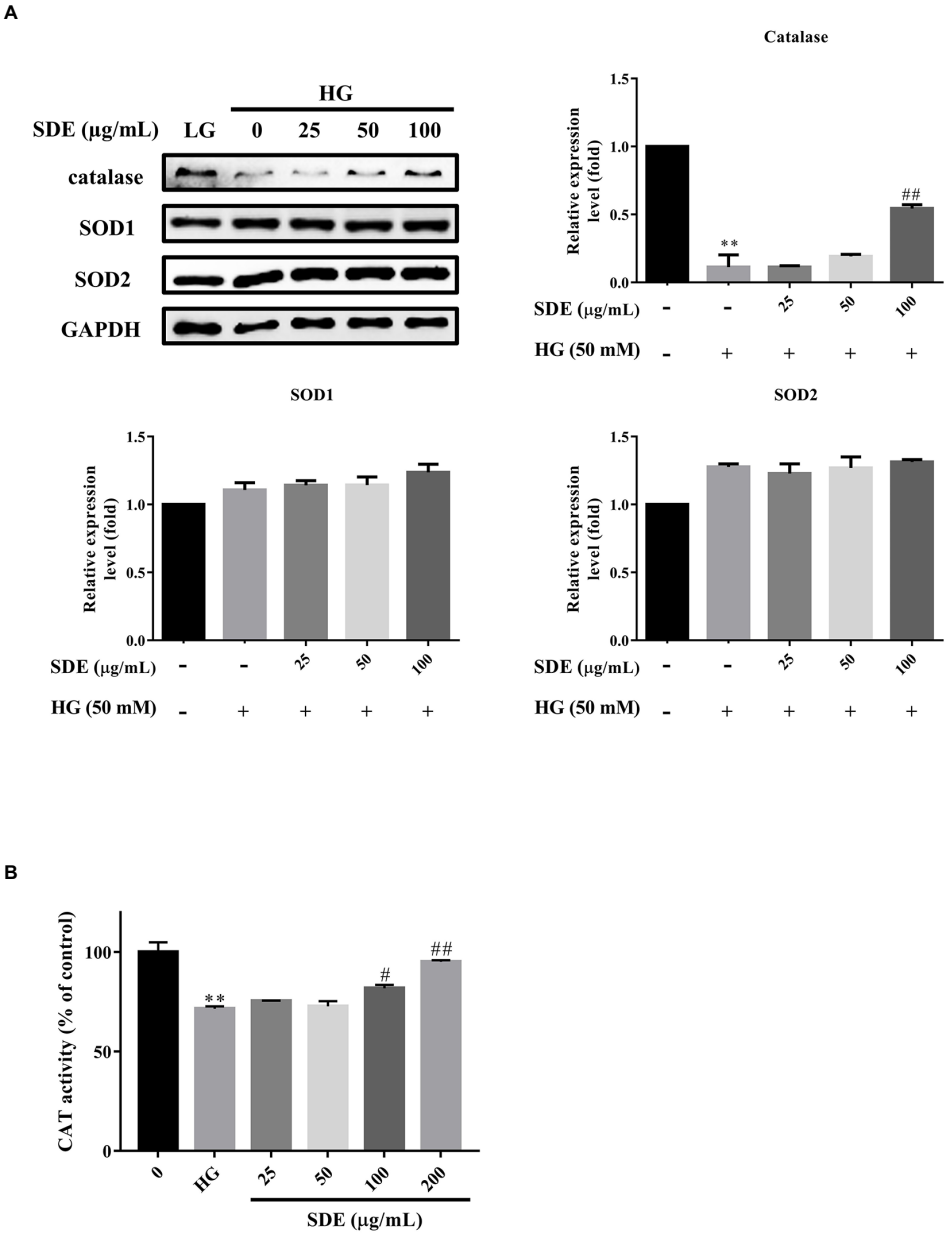
Effect of *Scoparia dulcis* L. extract (SDE) on anti-oxidative enzymes with high-glucose treatment on ARPE-19 cells. The ARPE-19 cells were treated with SDE (25, 50, 100 m or 200 μg/ml) for 24 h, and following this, they were treated with 50 mM glucose for 48 h. **(A)** The protein expression of anti-oxidative enzyme protein expression levels catalase SOD1 and SOD2 in cells with HG treatment was determined by Western blot analysis. **(B)** The catalase activity was determined by ELISA kit. *p < 0.05, **p < 0.01 compared with the control cells incubated in the low-glucose medium; ^#^*p* < 0.05, ^##^*p* < 0.01 compared with the ARPE-19 cells incubated in HG.

To investigate the anti-oxidative mechanisms of SDE against HG-induced cell damage, the protein expression levels of anti-oxidant enzymes including catalase, SOD1, and SOD2 were assessed by a Western blot analysis (Figure 4A). HG treatment significantly decreased the expression of catalase. In contrast, SDE enhanced catalase expression levels in a dose-dependent manner compared to the HG group. However, SOD1 and SOD2 seem to have demonstrated no significant change with SDE pretreatment. We further assessed the catalase activity to confirm the anti-oxidative effect of SDE (Figure 4B). As excepted, SDE was shown to be able to rescue catalase activity with HG treatment. These results indicate that catalase might be involved in the protective role of SDE against HG-induced oxidative stress.

### *Scoparia dulcis* L. extract induces the activation of Nrf2/HO-1 pathway in HG-stimulated ARPE-19 cells

**Figure 5:**
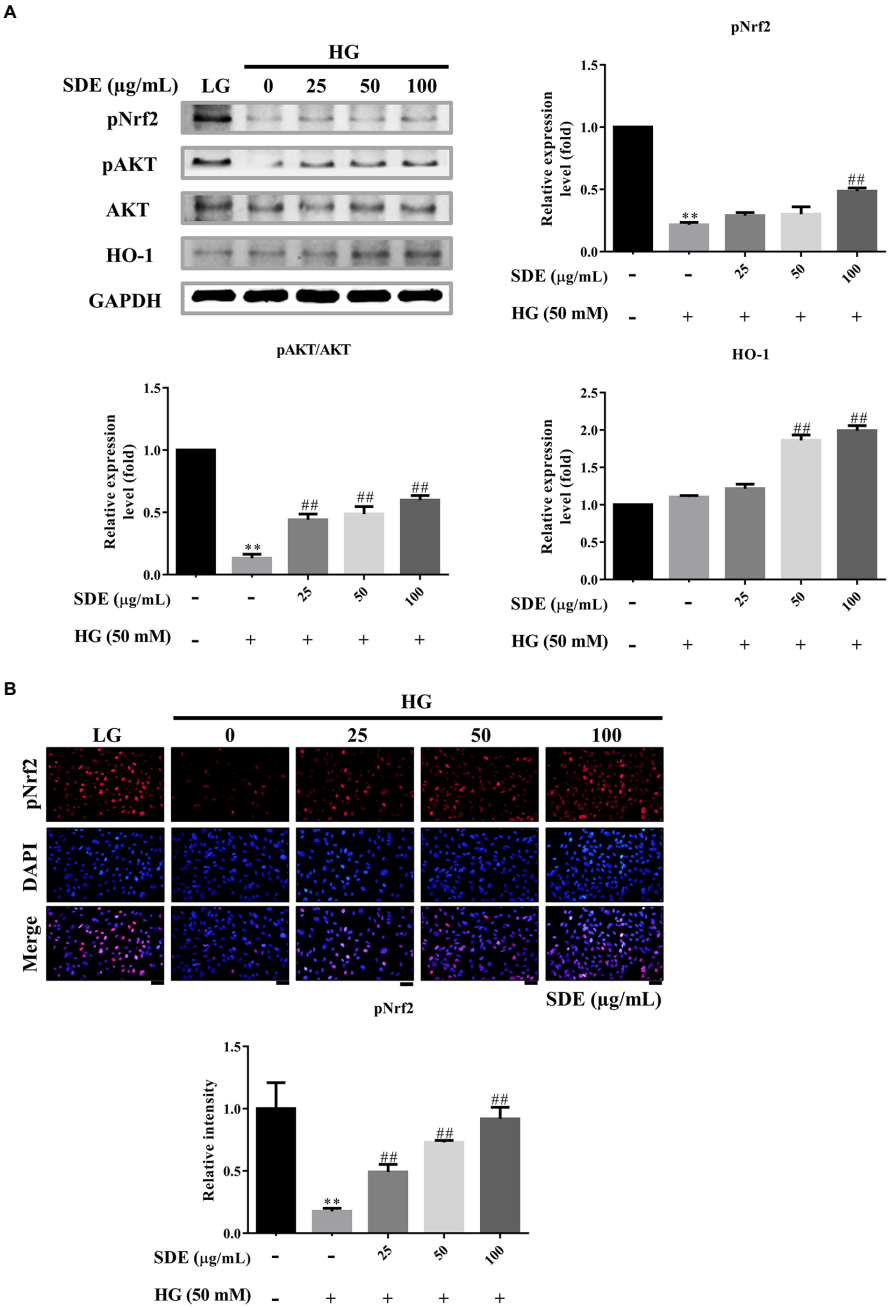
*Scoparia dulcis* L. extract (SDE) treatment inhibited high-glucose-induced inflammation, oxidative stress, and apoptosis by activating the Nrf2/ARE signaling pathway. After pretreatment with SDE (25, 50, 100 or 200 μg/ml) for 24 h, the ARPE-19 cells were incubated in a high-glucose (50 mM) medium for 48 h. **(A)** Protein expression of apoptosis-related protein expression levels pNrf2, pAKT, AKT, and HO-1 in cells induced by HG was tested by Western blot analysis. **(B)** pNrf2 were detected using immunofluorescence (IF) imaging. Scale bars, 50 μm. DAPI, 4′,6-diamidino-2- phenylindole; LG, low glucose; HG, high glucose; SDE, *Scoparia dulcis* L. extract. **p < 0.01 compared with the control cells incubated in the low-glucose medium; ^##^*p* < 0.01 compared with the ARPE-19 cells incubated in HG.

Phase II enzyme HO-1, which is a well-known anti-oxidant enzyme, reduces intracellular ROS. Furthermore, that the Nrf2/HO-1 pathway plays a critical role in the pathogenesis of diabetic retinopathy has been well documented (3). Hence, we examined the effect of SDE on Nrf2/HO-1 pathway activation in HG-stimulated ARPE-19 cells. We found that HG slightly stimulated HO-1 expression levels, and SDE significantly induced HO-1 expression. Moreover, SDE also rescued pNrf2 and pAKT activation compared to the HG group (Figure 5A). We further assessed pNrf2 activation by immunofluorescence staining. Figure 5B shows that the activation of Nrf2 was observed with SDE treatment. Our results indicate that SDE activates the Akt/Nrf2/HO-1 pathway, which further contributes to decreasing the ROS generated in a HG environment.

#### *Scoparia dulcis* L. extract inhibits the levels of pro-inflammatory cytokines in HG-stimulated ARPE-19 cells

**Figure 6:**
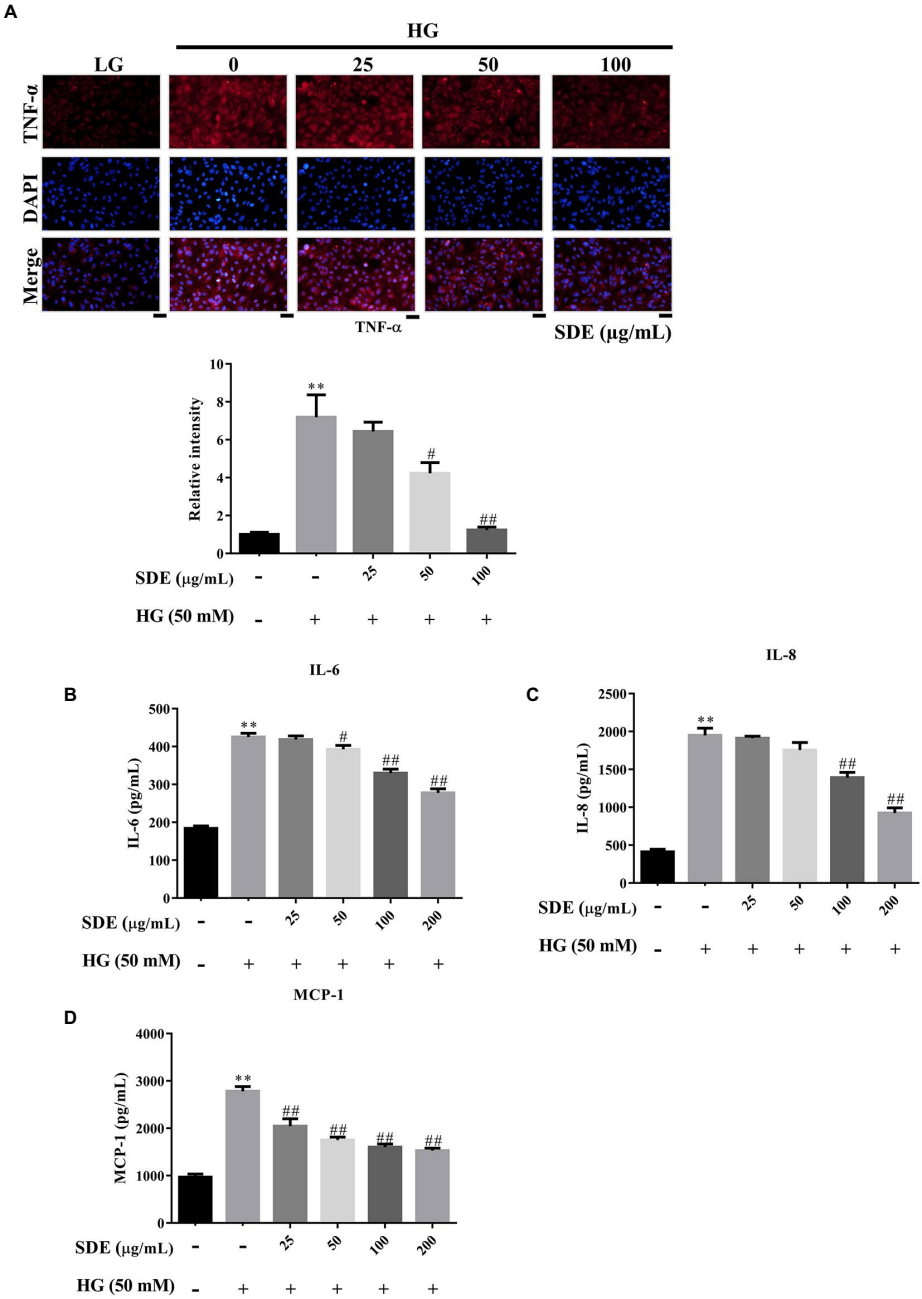
*Scoparia dulcis* L. extract (SDE) treatment alleviates cell inflammation in ARPE-19 cells with high-glucose treatment. After pretreatment with SDE (25, 50, 100, or 200 μg/ml) for 24 h, the ARPE-19 cells were incubated in a high-glucose (50 mM) medium for 48 h. **(A)** TNF-α immunostaining was detected using immunofluorescence (IF) imaging. **(B)** IL-6, **(C)** IL-8, and **(D)** MCP-1 expression of inflammatory mediators in a cell culture medium was assessed using an ELISA kit. *p < 0.05, **p < 0.01 compared with the control cells incubated in the low-glucose medium; ^#^*p* < 0.05, ^##^*p* < 0.01 compared with the ARPE-19 cells incubated in the high-glucose medium. Scale bars, 50 μm. DAPI, 4′,6-diamidino-2- phenylindole; LG, low glucose; HG, high glucose; SDE, *Scoparia dulcis* L. extract.

Our results have shown that SDE significantly increased AKT activation (Figure 5A), and pAKT has previously been associated with inhibition of the NF-κB inflammatory pathway (25). To further evaluate the effect of SD extract on pro-inflammatory cytokines production, we performed ELISA and immunofluorescence staining analysis. We found that SDE treatment dose-dependently inhibited the expression of tumor necrosis factor-α (TNF-α) (Figure 6A) and decreased the secretion of IL-6 (Figure 6B), IL8 (Figure 6C), and MCP-1 (Figure 6D) in the ARPE-19 cells exposed to HG. These results imply an anti-inflammatory effect of SD extract on HG-stimulated ARPE-19 cells.

## Discussion

In recent years, *in vitro* cultures of human RPE cells have presented an attractive model for studying the physiology and pathophysiology of diabetic retinopathy (26, 27). The human RPE cell line ARPE-19 exhibits epithelial cell morphology and expresses several genes specific to the RPE, which has been widely used as an alternative to primary RPEs and as an important model to study oxidative stress (28–30). However, ARPE-19 cells lose their RPE characteristics, such as the cobblestone appearance, polarity, and expression of RPE markers, after a few passages in the culture (31). Recently, some studies have shown that media conditions and length of culture time allow ARPE-19 cells to obtain a more native, physiological state. In the present study, the ARPE-19 cells grown in specialized differentiation DMEM media for 6 months were compared to cells grown in standard DMEM/F12 media. As observed, cells differentiated for 6 months exhibited tight junction protein (ZO-1) and RPE-specific markers (RPE-65) expression (Supplementary Figure S2A). Furthermore, the RPE-65 expression level was also examined in the cells by Western blotting (Supplementary Figure S2B). According to the results (Supplementary Figure S2), we used the 6-month ARPE-19 to establish an *in vitro* diabetic retinopathy model, which showed the possibility of obtaining a more differentiated physiologically native RPE state. However, these differentiated cells did not reach the pigmentation observed in other studies; this may be due to different factors such as the passage number, the surface substrate they are grown on, and the media used.

Many studies show that oxidative stress and inflammation play a critical role in ophthalmic diseases (5, 6). Hyperglycemia can induce oxidative stress mainly through flux of the polyol pathway and hexosamine pathway induction, the hyperactivation of protein kinase C (PKC) isoforms, and the accumulation of advanced glycation end products (AGEs) (32, 33). On the other hand, hyperglycemia can repress the anti-oxidant defense system *via* epigenetic modification, resulting in an imbalance between the scavenging and production of ROS (34) Finally, excessive accumulation of ROS induces mitochondrial dysfunction, lipid peroxidation, cellular apoptosis, inflammation, and structural or functional alterations in retina (3, 35). Hence, it is critical to investigate and elucidate the oxidative stress-related mechanisms of DR, which may provide multiple potential therapeutic targets to develop safe and effective treatments for DR.

*Scoparia dulcis* L. has been found to possess many pharmacological applications and biological activities, including anti-oxidative, anti-inflammatory, and anti-diabetic effects (17). To date, approximately 160 compounds have been identified from S. dulcis. Among them, 115 compounds may be related to the treatment of metabolic syndrome. Flavonoids and phenolics are the most important compounds in *Scoparia dulcis* L. In the present study, we identified the active compound of SDE, in accordance with the previous studies (19). Three compounds were identified, and the specific information of the compounds has been shown in Supplementary Table S2. β-cyclocitral is a main apocarotenoid of β-carotene, which are not only vitamin A components but also natural pigments and anti-oxidants (36). They are also involved in anti-oxidant signaling *via* the upregulation of various anti-oxidative enzymes such as superoxide dismutases (SOD), catalase (CAT), and peroxidase (POD) (37). Despite the function of β-cyclocitral in animals remaining unknown, β-cyclocitral-induced anti-oxidative activity may play a role as a bioactive compound in SDE. On the other hand, acetylspermine belongs to the polyamines family, and it plays an essential role in the proliferation and development of mammalian cells. Moreover, polyamines have been shown to protect against oxygen radical-mediated damage or to serve as substrates for oxidation reactions that produce hydrogen peroxide (H_2_O_2_) (38). There has been no anti-oxidant activity or anti-inflammation activity recorded by 1-methyl-2-pyrrolidinone. Our study also indicated that SDE inhibits HG-induced apoptosis (Figure 1C) and inflammatory cytokines (Figure 6). The possible reason for the protective effects of SDE against oxidative stress and inflammation in ARPE-19 cells may be because it is rich in natural anti-oxidants.

In this study, we demonstrated that SDE inhibits HG-induced oxidative stress, inflammation, and cell apoptosis or senescence. As we know, ROS is involved in diabetes and its complications (39). Several studies have indicated that oxidative stress is one critical contributor to the pathogenesis of diabetic retinopathy (3). High oxygen consumption and an active metabolism are essential to the visual imaging function in the retina, which leads to the accumulation of ROS and promotes lipid peroxidation (40). Our results suggest that SDE extract treatment could reduce intracellular ROS (Figure 2A) and mitochondria superoxide production (Figure 2B) through induced anti-oxidative enzyme expression, such as catalase and HO-1 (Figures 4, 5). The aqueous extract of *Scoparia dulcis* L. can significantly reduce plasma lipid peroxidation and enhance CAT, SOD, glutathione peroxidase (GPx), and glutathione-S-transferase (GSH) activity in STZ-induced diabetic rats, and this was shown to be slightly better than glibenclamide treatment (21). To further reveal the underlying mechanism of the cytoprotective activity of SDE, Nrf2 activation and the expression of downstream anti-oxidant enzymes were investigated. Nrf2 is a redox-sensitive transcription factor that regulates many phase II anti-oxidant enzyme expressions, and the activation of Nrf2 is proved to be one of the critical defensive mechanisms against oxidative stress (41, 42). There is increasing interest in the involvement of Nrf2/HO-1 in the progression of diabetic complications (41). Moreover, there has been considerable interest in developing Nrf2 activators for the treatment of diabetic retinopathy (42). It has been reported that Polygonatum sibiricum polysaccharides (PSP), an important component of Polygonatum sibiricum (PS) with anti-diabetic activity, promote Nrf2 and HO-1 expression in ARPE-19 cells (26). In line with their study, we also demonstrated that SDE treatment enhances the HG-induced activation of the Nrf2/HO-1 signaling pathway.

The aqueous extract of *Scoparia dulcis* L. exhibited an anti-inflammatory potential effect in the carrageenan or dextran-induced rat paw edema model (43, 44). There is no denying that pro-inflammatory cytokines/chemokines play an essential role in its pathogenesis, and many studies have indicated that anti-inflammatory medication could delay early DR (45–47). TNF-α has been previously detected in the retinas of diabetic rats and DR patients (24). Inhibition of TNF-α has been reported to suppress NF-κB activation and BRB breakdown (48). Induction of MCP-1 can induce the production of superoxide and other mediators, which leads to capillary obstruction, vascular leakage, and absence of perfusion in the pathogenesis of DR (27). Previous studies have demonstrated that IL-6, IL-8, and MCP-1 not only initiate inflammatory responses but also promote angiogenesis, thereby stimulating DR progression (49–51). In addition, it has been reported that HG induces the secretion of inflammatory cytokines in ARPE-19 cells (52). In the present study, we found that SDE alleviates IL-6, IL-8, and MCP-1 levels (Figures 6B,D), and we also found that SDE alleviates TNF-α expression (Figure 6A), which was commonly observed in DR tissues (53) These results indicate that SDE may be a potential therapeutic agent for diabetes mellitus treatment.

There were a few limitations to our study. First, we treated the ARPE-19 cells with SDE before the high-glucose administration; therefore, we evaluated only the effects of SDE on the acute response of cells to high glucose but not on the cells that had already developed a certain degree of damage under the high-glucose environment. Previous *in vivo* studies have demonstrated that SDE administered after the induction of diabetes animal models can reduce the levels of plasma lipid peroxidation and enhance anti-oxidant enzyme activity (21). These results indicate that SDE may potentially be used for the treatment of high-glucose-induced damage, but further animal studies are needed to confirm the cellular responses of SDE administered after the occurrence of cellular damage. Second, we only used the CCK-8 and TUNEL assays to determine HG-induced cell damage. Nevertheless, we used other ancillary tests, such as β-Galactosidase staining for cell senescence and immunostaining for oxidative lipid or protein products, to support our conclusions.

## Conclusion

In this study, we have demonstrated that SDE could protect ARPE-19 cells from apoptosis and senescence by inhibiting HG-induced oxidative stress and inflammation. In addition, we have demonstrated that the protective effects of SDE are mediated by the activation of AKT and Nrf2, which increase the expression of downstream phase II enzymes or anti-oxidative enzymes. Thus, SDE should be considered as a nutritional supplement that could benefit patients with diabetes, especially in preventing visual loss in DR.

## Data availability statement

The original contributions presented in the study are included in the article/supplementary material, further inquiries can be directed to the corresponding author/s.

## Author contributions

H-DL: study design, data collection, data analysis, writing the manuscript. Y-CL: study design, data analysis. C-YC: data collection, data analysis. Y-JL: data collection, preparing the figures. CS: study design, material support. R-KT: study design, data analysis. P-YL: study design, material support. S-ZL: study supervision, reviewing the manuscript. T-JH: data analysis, reviewing the manuscript. C-YH: reviewing the final version of the manuscript. All authors contributed to the article and approved the submitted version.

## Funding

This work was supported by grants from the Hualien Tzu Chi Hospital, Buddhist Tzu Chi Medical Foundation (IMAR-111-01-08), in Taiwan.

## Conflict of interest

The authors declare that the research was conducted in the absence of any commercial or financial relationships that could be construed as a potential conflict of interest.

## Publisher’s note

All claims expressed in this article are solely those of the authors and do not necessarily represent those of their affiliated organizations, or those of the publisher, the editors and the reviewers. Any product that may be evaluated in this article, or claim that may be made by its manufacturer, is not guaranteed or endorsed by the publisher.
