# Supplementary material for: Protective effects of Scoparia dulcis L. extract on high glucose-induced injury in human retinal pigment epithelial cells
Source: Front Nutr. 2023 Mar 30;10:1085248. doi: 10.3389/fnut.2023.1085248 (PMC10150881; doi:10.3389/fnut.2023.1085248)
Supplement: Supplementary file 1 [file Table_1.DOCX]

**Table S1.** List of primary and secondary antibodies used for western blot (WB) and Immunofluorescence (IF).

| **Primary Antibodies** | **Company** | **Catalog number** | **Dilution** |
| --- | --- | --- | --- |
| catalase | Santa Cruz | sc-271803 | 1:1000 (WB) |
| SOD1 | Santa Cruz | sc-17767 | 1:1000 (WB) |
| SOD2 | Santa Cruz | sc-133134 | 1:1000 (WB) |
| pNRF2 | abcam | ab76026 | 1:1000 (WB) |
| pNRF2 | abcam | ab76026 | 1:200 (IF) |
| AKT | Santa Cruz | sc-5298 | 1:1000 (WB) |
| pAKT | Santa Cruz | sc-514032 | 1:1000 (WB) |
| HO-1 | Santa Cruz | sc-136961 | 1:1000 (WB) |
| RPE65 | Abcam | Ab231782 | 1:1000 (WB) |
| RPE-65 | Santa Crus | Sc-390787 | 1:200 (IF) |
| ZO-1 | Abcam | ab221547 | 1:200 (IF) |
| Acrolein | Abcam | Ab240918 | 1:200 (IF) |
| 3-Nitrosine | Abcam | ab110282 | 1:200 (IF) |
| TNF-α | Santa Cruz | sc-52746 | 1:200 (IF) |
| **Secondary Antibodies** | **Company** | **Catalog number** | **Dilution** |
| mouse anti-rabbit IgG-HRP | Santa Cruz | sc-2357 | 1:3000 (WB) |
| mouse-IgGκ BP-HRP | Santa Cruz | sc-516102 | 1:3000 (WB) |
| 488 goat anti-mouse IgG (H+L) | Invitrogen | A11001 | 1:400 (IF) |
| 594 goat anti-mouse IgG (H+L) | Invitrogen | A11032 | 1:400 (IF) |
| 488 goat anti-rabbit IgG (H+L) | Invitrogen | A11008 | 1:400 (IF) |
| 594 goat anti-rabbit IgG (H+L) | Invitrogen | A11012 | 1:400 (IF) |
