# Supplementary material for: Protective effects of Scoparia dulcis L. extract on high glucose-induced injury in human retinal pigment epithelial cells
Source: Front Nutr. 2023 Mar 30;10:1085248. doi: 10.3389/fnut.2023.1085248 (PMC10150881; doi:10.3389/fnut.2023.1085248)
Supplement: Supplementary file 2 [file Table_2.DOCX]

**Table S2.** Bioactive components identified in SDE.

| Analyte Name | β-Cyclocitral | 1-Methyl-2- Pyrrolidinone | N1- Acetylpermine |
| --- | --- | --- | --- |
| SDE-1 | 0.3315 | 0.0152 | 0.5100 |
| SDE-2 | 0.1955 | 0.0143 | 1.3900 |
| SDE-3 | 0.2815 | 0.0167 | 0.5000 |

Mass concentration (µg/mL)
