# Supplementary figures and images for: Protective effects of Scoparia dulcis L. extract on high glucose-induced injury in human retinal pigment epithelial cells
Source: Front Nutr. 2023 Mar 30;10:1085248. doi: 10.3389/fnut.2023.1085248 (PMC10150881; doi:10.3389/fnut.2023.1085248)

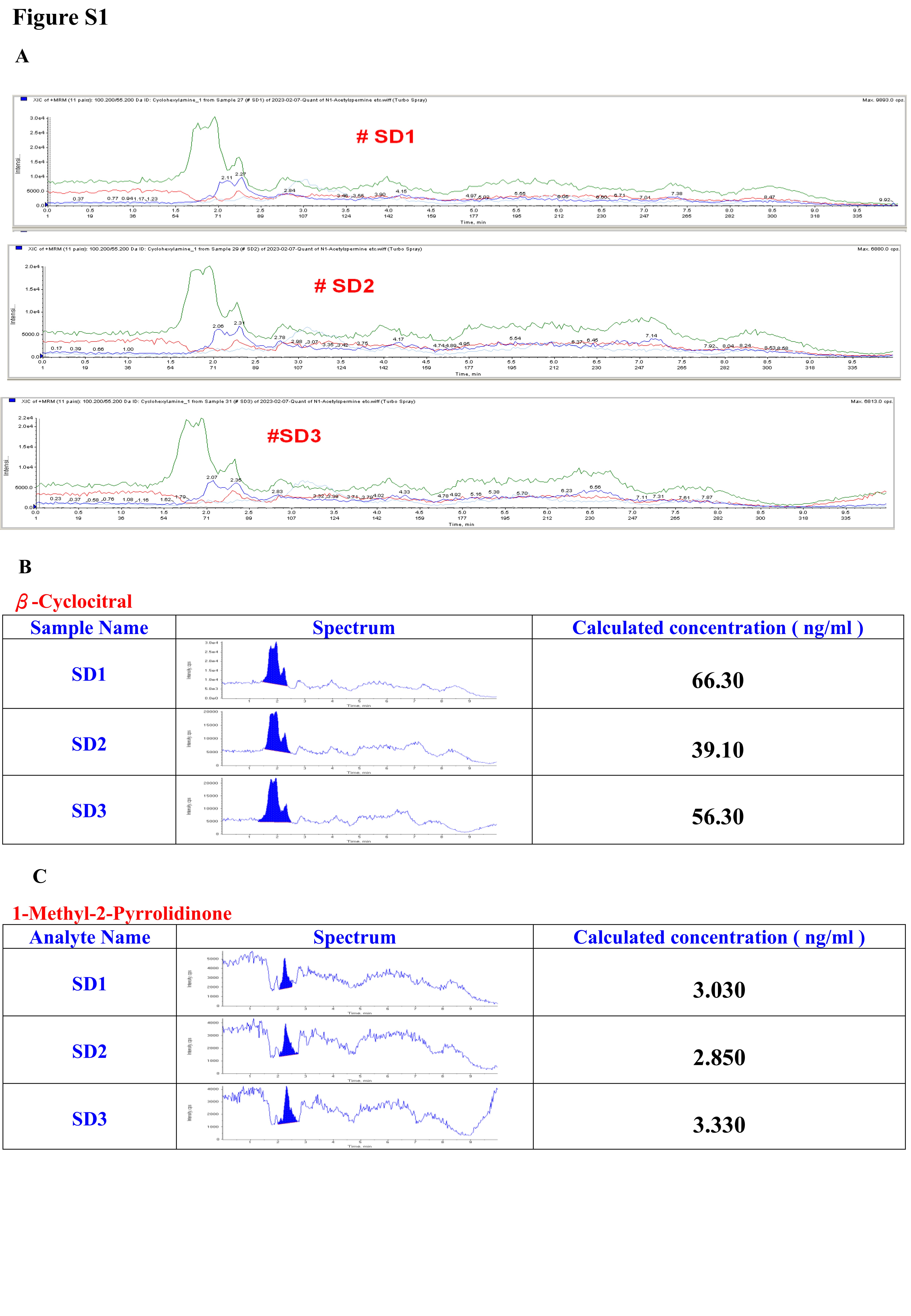

Supplement: Supplementary file 3 [file Image_1.JPEG]

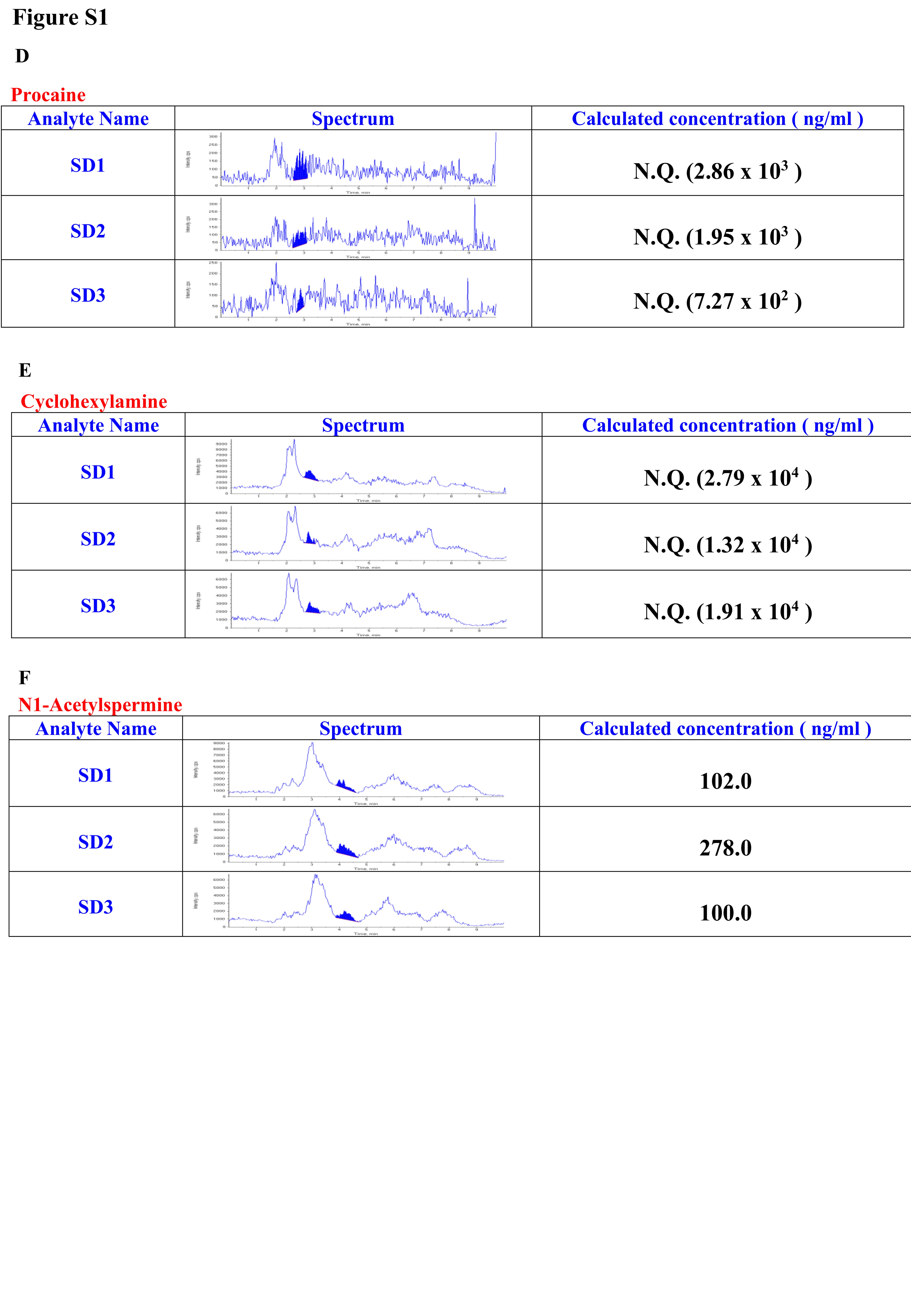

Supplement: Supplementary file 4 [file Image_2.JPEG]

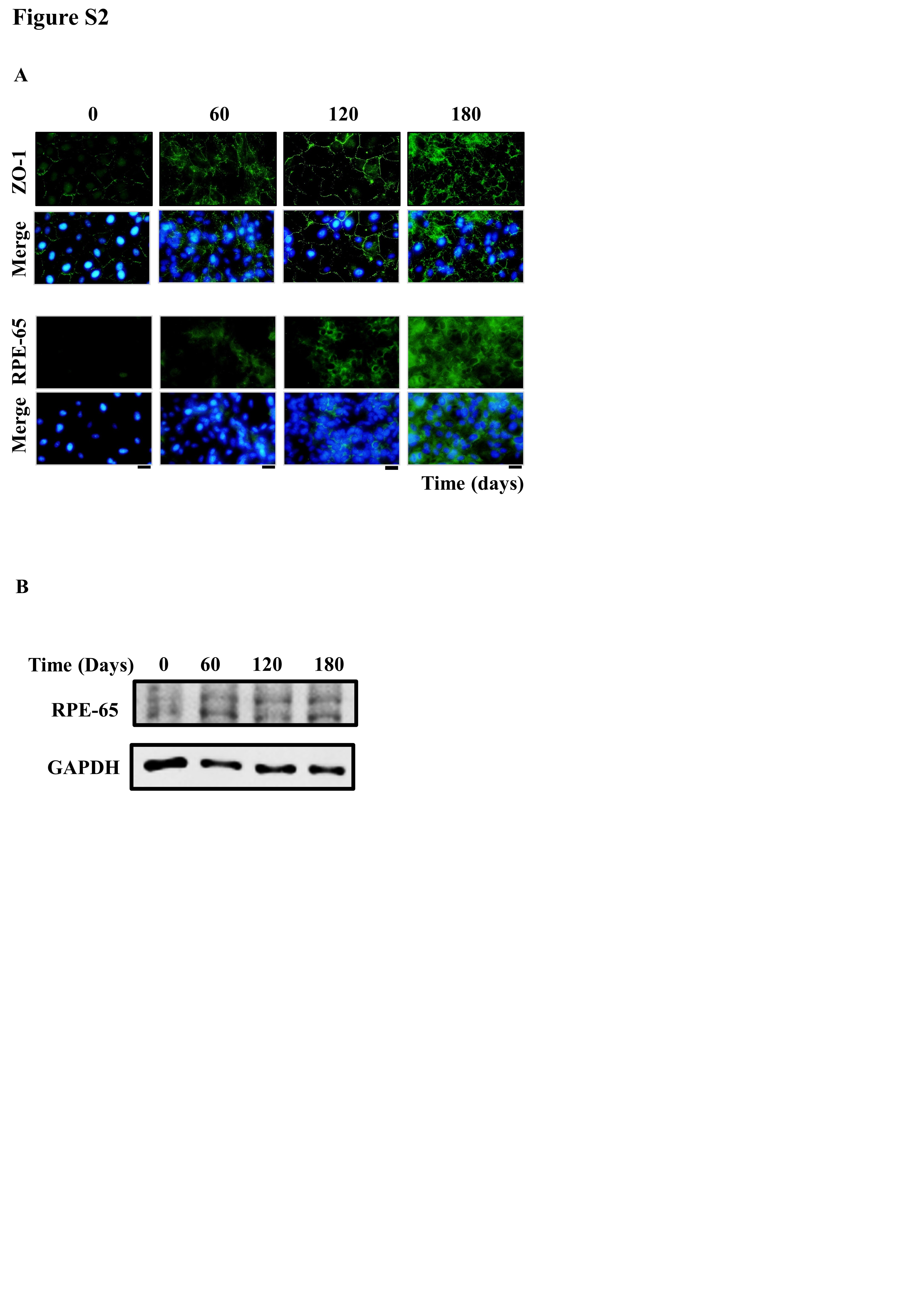

Supplement: Supplementary file 5 [file Image_3.JPEG]
